# Symptom Severity, Infection Progression and Plant Responses in Solanum Plants Caused by Three Pospiviroids Vary with the Inoculation Procedure

**DOI:** 10.3390/ijms22126189

**Published:** 2021-06-08

**Authors:** Francisco Vázquez Prol, Joan Márquez-Molins, Ismael Rodrigo, María Pilar López-Gresa, José María Bellés, Gustavo Gómez, Vicente Pallás, Purificación Lisón

**Affiliations:** 1 Instituto de Biología Molecular y Celular de Plantas (IBMCP), Consejo Superior de Investigaciones Científicas (CSIC), Universitat Politècnica de València (UPV), Ciudad Politécnica de la Innovación (CPI) 8 E, Ingeniero Fausto Elio s/n, 46011 Valencia, Spain; fravazpr@ibmcp.upv.es (F.V.P.); joama13j@posgrado.upv.es (J.M.-M.); irodrig@ibmcp.upv.es (I.R.); mplopez@ceqa.upv.es (M.P.L.-G.); jmbelles@btc.upv.es (J.M.B.); vpallas@ibmcp.upv.es (V.P.); 2 Institute for Integrative Systems Biology (I2SysBio), Consejo Superior de Investigaciones Científicas (CSIC), Universitat de València (UV), Parc Científc, Cat. Agustín Escardino 9, 46980 Paterna, Spain; gustavo.gomez@csic.es

**Keywords:** Pospiviroidae, CEVd, TCDVd, PSTVd, agro-infiltration, transcript, eggplant, tomato, ribosome, stress

## Abstract

Infectious viroid clones consist of dimeric cDNAs used to generate transcripts which mimic the longer-than-unit replication intermediates. These transcripts can be either generated in vitro or produced in vivo by agro-inoculation. We have designed a new plasmid, which allows both inoculation methods, and we have compared them by infecting *Solanum lycopersicum* and *Solanum melongena* with clones of *Citrus exocortis virod* (CEVd), *Tomato chlorotic dwarf viroid* (TCDVd), and *Potato spindle tuber viroid* (PSTVd). Our results showed more uniform and severe symptoms in agro-inoculated plants. Viroid accumulation and the proportion of circular and linear forms were different depending on the host and the inoculation method and did not correlate with the symptoms, which correlated with an increase in *PR1* induction, accumulation of the defensive signal molecules salicylic (SA) and gentisic (GA) acids, and ribosomal stress in tomato plants. The alteration in ribosome biogenesis was evidenced by both the upregulation of the tomato ribosomal stress marker *SlNAC082* and the impairment in 18S rRNA processing, pointing out ribosomal stress as a novel signature of the pathogenesis of nuclear-replicating viroids. In conclusion, this updated binary vector has turned out to be an efficient and reproducible method that will facilitate the studies of viroid–host interactions.

## 1. Introduction

Viroids are the simplest pathogens with autonomous replication currently known, and have only been found naturally in higher plants [1]. These covalently closed small single-stranded RNAs (246–401 nt) are the causal agents of several plant diseases, with a symptomatology similar to that associated with some plant viruses [2,3,4]. However, in many cases, viroid infection is asymptomatic or causes mild symptoms [5,6].

Due to the extreme simplicity of their circular RNA genome, viroids extensively rely on host factors for their movement and replication [7,8]. Furthermore, they can exert wide transcriptional changes in host plants, interfering with the silencing machinery of the cell [9,10], or producing epigenetic modifications [11,12,13], alternative splicing [14,15] or, as described recently, ribosomal stress [16,17]. In particular, this latter phenomenon refers to the impaired processing of the 18S rRNA provoked by the viroid, which causes defects in ribosome biogenesis, and important alterations in the host translation machinery [16,17,18]. Moreover, viroids are able to trigger the plant’s defense response, inducing the expression of pathogenesis related (PR) proteins, such as PR1 [18,19], and the accumulation of defensive signal molecules, including SA and GA [20,21,22]. However, this defense response activation is not efficient enough to combat the viroid infection, correlating PR1 induction and SA and GA accumulation with viroid symptomatology [20,21,22]. 

The classification of viroids consists of two families: Avsunviroidae and Pospiviroidae, and most currently known viroids belong to the latter [23,24]. Members of the Pospiviroidae family are characterized by a rod-like secondary structure with a central conserved region (CCR) and a terminal conserved hairpin (TCH) or a terminal conserved region (TCR), nuclear replication by an asymmetrical rolling circle, and, generally, a broad host range [25,26,27,28]. Among the hosts of nuclear-replicating viroids, the Solanum genus comprises some of the most affected species [29]. Tomato (*Solanum lycopersicum*) has been commonly used as an experimental host of several viroids, and the first viroid was discovered precisely in potato (*Solanum tuberosum*) [30]. Additionally, other crops of this genus, like eggplant *(Solanum melongena*), which is usually grown in tightly-packed greenhouse conditions, can also be infected by viroids of the Pospiviroidae family [31,32]. The symptoms caused by viroid infection may affect crop quality and/or yield, and are dependent on viroid species and strain, plant cultivar, and temperature, which could be due to the role of RNA silencing in viroid pathogenesis [33,34,35]. 

In order to produce viroid infections in the laboratory, dimeric cDNA clones of the entire viroid sequence are used to generate transcripts which mimic the longer-than-unit replication intermediates and, therefore, can be processed into unitary, circular RNAs within the cell [36,37]. The first viroid cDNA infectious clone was developed in 1983 [36] and since then, constructs of this type have been used to study viroid pathogenesis in natural and experimental hosts. The use of these clones instead of nucleic acids extracts from infected plants has several advantages. First, traditional methods using sap extracts or grafting procedures are often inefficient and non-reproducible. Moreover, cDNA of viroid dimers provides a near-homogenous inoculum, whereas if isolated from infected plants the viroid RNA consists of a mix of sequence variants. Finally, directed modifications in the viroid sequence can be performed, which have enabled the identification of motifs or domains that are important for viroid replication and/or movement [38,39,40,41], and also the characterization of a domain that interacts with a certain host factor [42]. These dimeric RNAs of the viroid can be delivered by inoculation with in vitro generated transcripts or can be produced in vivo by agrobacterium-mediated transient plant transformation. Until recently, dimeric clones of viroids were constructed by self-ligation of monomeric cDNA in presence of T4 DNA ligase [36,43]. Nonetheless, a new method based on IIS restriction enzymes enables a more efficient way and straightforward construction of viroid dimers [44]. 

CEVd [45], PSTVd [46] and TCDVd [47] are pospiviroids that replicate in *Solanum* plants producing evident phenotypic alterations [16,47]. To evaluate the influence of the inoculation method on the viroid infection progress in *Solanum* hosts, we have developed infectious clones of these three pospiviroids in an updated binary vector which allows both agro-infiltration, and a more efficient in vitro transcription of the dimeric viroid cDNA. Several aspects of the viroid infection process such as symptomatology, viroid titre, activation of the plant defense response, and alterations in the ribosomal biogenesis are studied to evaluate the efficiency of the inoculation method.

## 2. Results

### 2.1. pMD201t2: A Highly Efficient Plasmid for Both In Vitro Transcription and In Vivo Agro-Inoculation 

**Figure 1:**
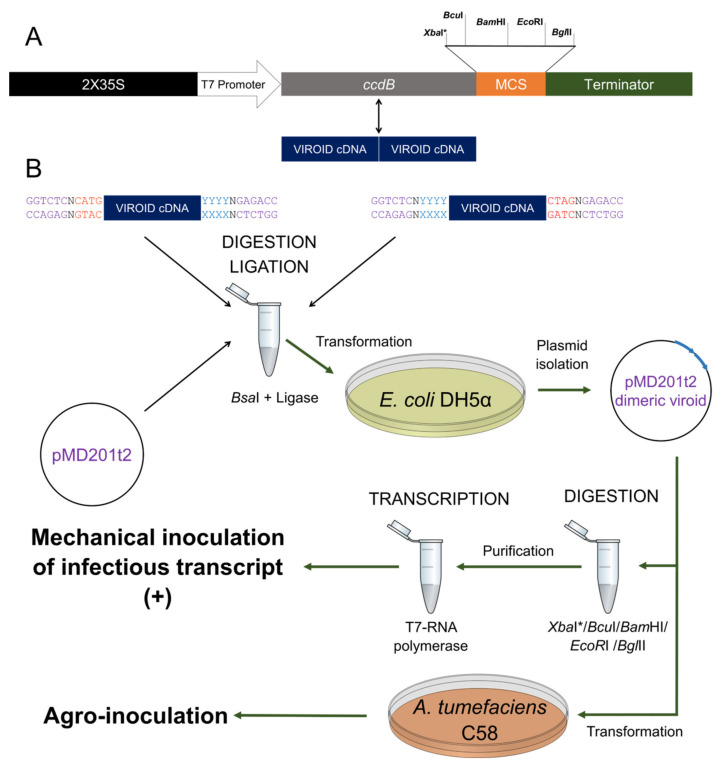
Generation of head-to-tail dimeric viroid cDNAs into pMD201t2. (**A**) Scheme of the transcriptional unit (TU) designed for generating dimeric viroid RNAs in vitro or in vivo by agro-inoculation. This TU which consists of a duplicated 35S followed by a T7 promoter, the lethal gene *ccdB* and the PoPit terminator preceded by a multiple cloning site (MCS). In the cloning process, the dimeric viroid cDNA sequence replaces the lethal gene, therefore being a highly efficient reaction. (**B**) Viroid sequences are amplified with two pairs of primers containing the *Bsa*I recognition site (purple) designed to generate compatible ends between the viroid cDNA (blue) and to the plasmid (red). The dimeric viroidal cDNA cloned into pMD201t2 can be used to generate the infectious RNA transcript in vitro, using T7 RNA polymerase onto a linearized plasmid (digested with *Bcu*I, *Bam*HI, *Eco*RI *Bgl*II, and *Xba*I*). Additionally, the pMD201t2-viroid can be transformed into *Agrobacterium tumefaciens* for transient plant transformation and subsequent production of infective viroid RNAs in vivo. * If the viroid cDNA sequence ends in T.

Taking the binary vector pMD201t [44] as a starting point, we designed a new configuration based on this backbone to optimize in vitro transcription (Figure 1A). This new vector (pMD201t2) has three modifications in comparison with its predecessor: (i) the size was reduced from 11558 to 9437 pb (10093 and 7972 pb, respectively, before and after the excision of the lethal gene by *Bsa*I digestion) (ii) the localization of the T7 promoter for in vitro transcription was changed in order to avoid the transcription of the duplicated 35S promoter (iii) a multicloning-like site was introduced between the cloning site for the viroid and the terminator sequence in order to linearize the plasmid, which is necessary for in vitro transcription (Figure 1A). Thus, a minimum of four different restriction sites are present, and five in case the viroid cDNA ends in a T residue (Figure 1A). No deposited viroid sequence in publicly available databases simultaneously contains the four internal recognition sites (Table S1) and therefore infectious transcripts of any viroid can be potentially generated with this vector.

Constructs of head-to-tail dimeric viroid of PSTVd, TCDVd and CEVd were cloned into pMD201t2. PSTVd cDNA was amplified from a previous generated plasmid [48], while monomeric TCDVd was directly synthesized, and CEVd was amplified by RT-PCR from infected tissue. The amplification was performed with two pairs of primers that contain the *Bsa*I recognition site (Figure 1B), designed to generate compatible ends upon digestion. The resulting monomeric cDNAs were purified and a reaction of digestion-ligation was set up and transformed into *E. coli* DH5a. In all cases, the efficiency of the dimerization into the binary vector was 100%, as the lethal gene *ccdB* results in a zero-background selection [44]. On the one hand, the binary vectors harboring the viroid dimers were transformed into *Agrobacterium tumefaciens*. On the other hand, these same plasmids were linearized and transcribed in vitro.

### 2.2. Influence of the Inoculation Method on the Symptomatology and Viroid Accumulation in Solanum Lycopersicum and Solanum Melongena

**Figure 2:**
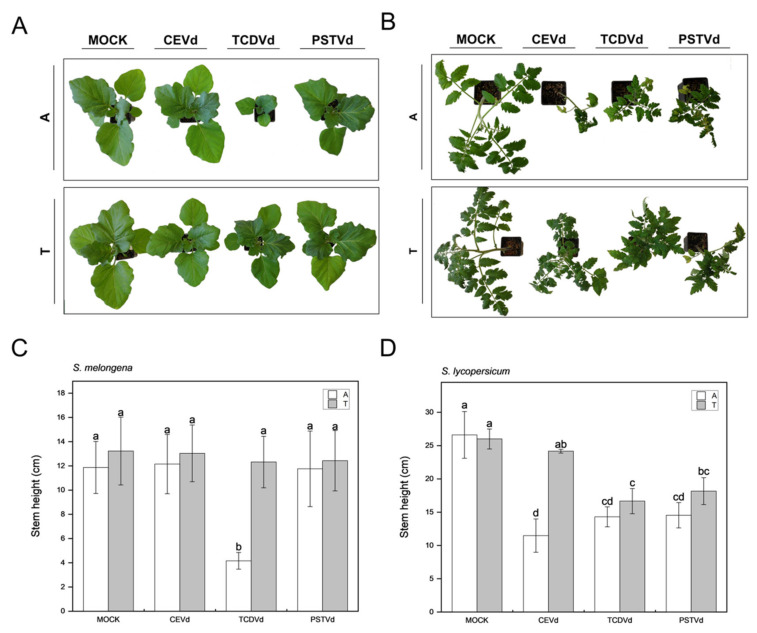
Symptomatology caused by CEVd, TCDVd, and PSTVd by agro- and transcript-inoculations in eggplant and tomato. Representative images of *Solanum melongena* cv Black Beauty (**A**) and *Solanum lycopersicum* cv Rutgers (**B**) of mock and viroid-inoculated plants using agro-inoculation (upper images; A) and transcript (lower images; T). (**C**) Quantitation of mean leaf size in agro- and transcript-inoculated eggplant plants. (**D**) Quantitation of stem height in agro- and transcript-inoculated tomato plants. Data correspond to the mean plants ± SD of at least three independent replicates. Data were analyzed using a Mann–Whitney test and different letters indicate significant differences (*p ≤* 0.05).

Plants in the cotyledon stage of *S. lycopersicum* and *Solanum melongena* were inoculated either with the in vitro generated dimeric transcripts, or with the different *Agrobacterium* harboring the constructs to produce viroid dimeric RNAs. In general terms, phenotypic alterations such as leaf rugosity and stunting were evident in all tomato plants inoculated with CEVd, TCDVd, or PSTVd (Figure 2B), whereas only eggplants agro-inoculated with TCDVd displayed stunting (Figure 2A). More severe specific symptoms such as epinasty in plants inoculated with CEVd and PSTVd, or chlorosis in those infected with TCDVd, were only observed in tomato plants.

To quantify differences between symptoms caused by transcript- and agro-inoculation methods, measures of the stem height in tomato (Figure 2C), and the width of expanded leaves in eggplant (Figure 2D) were performed. Tomato plants agro-inoculated with CEVd, PSTVd, and TCDVd showed shorter stems than non-infected plants, displaying statistically significant differences between both inoculation procedures in CEVd-infected plants (Figure 2D). In the case of eggplant, only plants infected with TCDVd showed significant differences between methods, agro-inoculated plants displaying narrower leaves.

To better evaluate the appearance and progression of phenotypic alterations in tomato plants, we monitored stem and internode length of agro-inoculated plants with each viroid, along the infection progress. The first visible symptoms appeared at 14 dpi in tomato plants infected by agro-inoculation with PSTVd. However, at 25 dpi, symptoms caused by CEVd were the most severe (Figure S1) as internode and stem length measures shown, thus indicating that the progression of the symptoms is different for each viroid.

**Figure 3:**
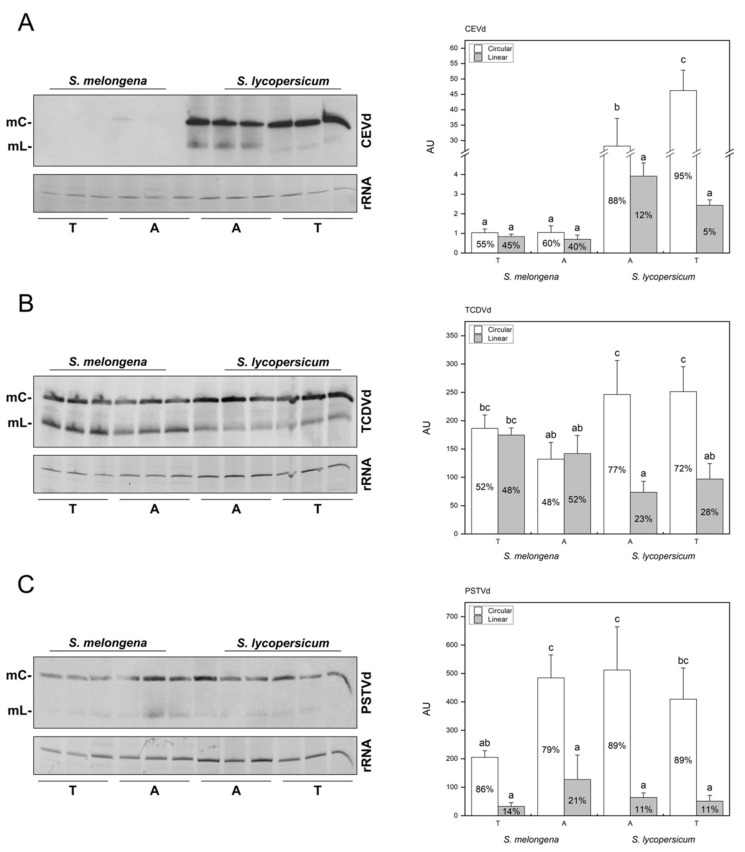
Viroid accumulation in *S. lycopersicum* and *S. melongena*. Northern blot assay and quantification for detecting plus polarity circular (mC) and linear (mL) viroid molecules in systemic tissue of transcript-inoculated plants (T) or by agro-inoculation and quantification of viroid accumulation in infected plants for CEVd (**A**), TCDVd (**B**), and PSTVd (**C**). Bars correspond to absolute AU values, and percentage of each form over total viroid values are printed inside the bars. Data correspond to the mean ± SD of 3 biological replicates. Results were analyzed using an ANOVA test with a Tukey post-hoc, and different letters indicate significant differences (*p* ≤ 0.05).

Northern blot assays with specific probes of the three viroids were used to estimate the accumulation levels of the viroids in each host, and the proportion of circular and linear forms, to compare the two inoculation methods (Figure 3). CEVd was barely detected as a circular form in eggplant in only one of the agro-inoculated samples, suggesting evident limitation for development of systemic infection in this viroid/host system (Figure 3A, left panel). Conversely, CEVd was detected in all tomato samples, displaying absolute levels of accumulation higher than those detected in eggplant for all the samples. Particularly, a higher proportion of mature circular forms in transcript-inoculated plants (95%) was detected when compared to the agro-inoculated ones (88%; Figure 3A, right panel). Regarding TCDVd, overall accumulation levels were similar in both hosts. However, the ratio of circular and linear forms differed drastically, with a similar amount of each form in eggplant but mainly circular forms in tomato (Figure 3B). PSTVd also accumulated efficiently in both hosts, but the circular form was the majority in all cases and the agrobacterium-based inoculation procedure seemed to be more efficient (Figure 3C).

In conclusion, tomato plants displayed stronger symptomatology and viroid accumulation for the three *Pospiviroidae* when compared to eggplants, and CEVd-agro-inoculated tomato plants displayed the most severe symptoms. PSTVd agrobacterium-based inoculation was observed to be more efficient than the transcript-based procedure, especially in eggplant.

### 2.3. Effect of the Inoculation Method on the Activation of the Plant Defence Response in Tomato Plants

**Figure 4:**
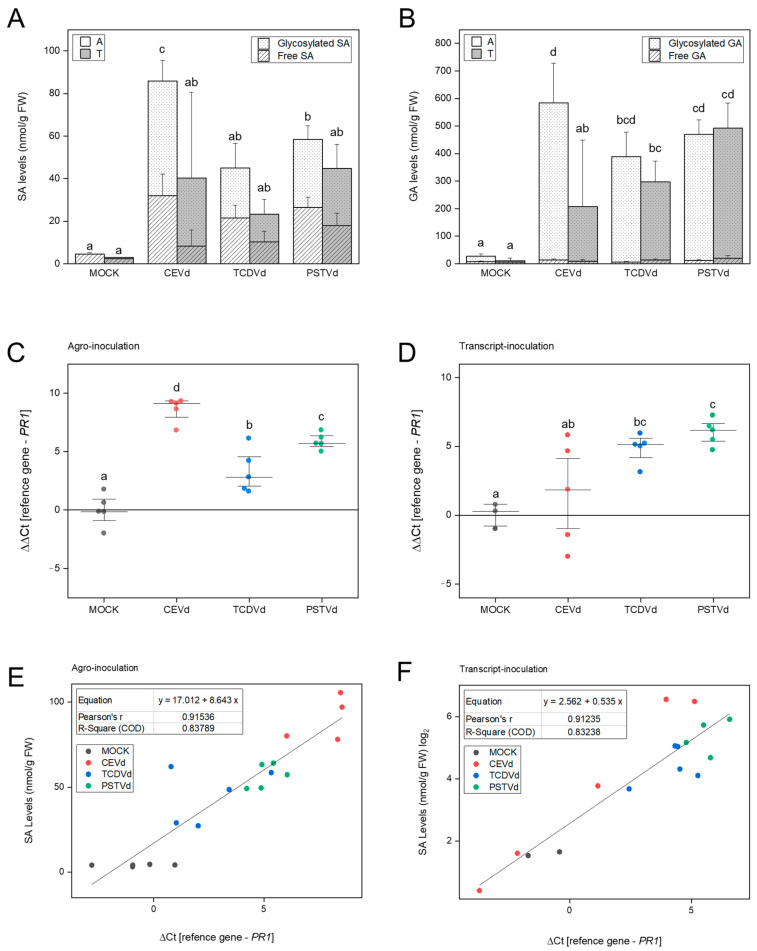
Activation of plant defense response in Rutgers tomato plants infected with CEVd, TCDVd, or PSTVd. (**A**) Levels of free and glycosylated SA and (**B**) GA in mock agro- and transcript-inoculated Rutgers leaves, respectively, infected with CEVd, TCDVd, and PSTVd at 25 days post inoculation (dpi). Data correspond to the mean ± SD of 5 biological replicates. Total levels of SA and GA were analyzed using an ANOVA test with a Tukey post-hoc and different letters indicate significant differences (*p* ≤ 0.05). Real-time PCR analysis of *PR1* expression in agro-inoculated (**C**) and transcript-inoculated (**D**) plants with mock, CEVd, TCDVd, and PSTVd at 25 dpi. Correlation value and equation between the relative induction of *PR1* and total SA levels in agro-inoculated (**E**) and transcript-inoculated (**F**) tomato plants. Data were analyzed using a Mann–Whitney test and different letters indicate significant differences (*p* ≤ 0.05).

Glycosylated and free SA and GA levels were analyzed in tomato plants infected by either transcript- or agro-inoculation at the end of the viroid infective process (25 dpi). Elevated total SA and GA accumulation was detected in CEVd-, TCDVd-, and PSTVd- agro-inoculated tomato leaves (Figure 4) when compared with the corresponding mock plants, which were agro-inoculated with the empty plasmid, GA levels being higher than those detected for SA. Significantly higher levels of total SA and GA were observed on tomato plants upon CEVd inoculation compared to the other viroids. This accumulation was also significantly higher in agro-inoculated plants when compared with those inoculated with transcript. These results correlate with the severity of the symptoms previously stated, thus indicating that the agro-inoculation method appears to be more efficient. Moreover, no statistical differences were observed when comparing SA and GA levels in transcript-inoculated plants to mock-inoculated plants, aside from GA levels in PSTVd-infected plants, and standard deviation was higher than in agro-inoculated plants for most samples. Regarding the glycosylation of these phenolics, we observed that about 55% of SA and more than 95% of GA accumulated in the infected leaves were in the conjugated form, with similar percentages in all viroids and inoculation methods.

To better characterize the plant defense response triggered by the three viroids upon both inoculation procedures, *PR1* gene expression was measured in the different infected tomato plants and a significant induction of this defense gene was observed in all the plants agro-inoculated with the viroid cDNAs when compared with the corresponding mock plants agro-inoculated with the empty plasmid, with the highest induction produced by CEVd (Figure 4C). Regarding transcript-inoculated plants, a high variability was observed in CEVd infected plants, and a significant induction of *PR1* was observed for the rest of the viroids (Figure 4D). Correlation values and equations were also calculated for SA levels and PR1 induction, and a clear correlation was observed in both transcript-inoculated and agro-inoculated plants (Figure 4E,F). Therefore, our results appear to indicate that CEVd agro-inoculated tomato plants appear to display the most severe symptoms, and the highest plant defense activation.

### 2.4. Induction of Ribosomal Stress Correlates with the Symptoms-Intensity

**Figure 5:**
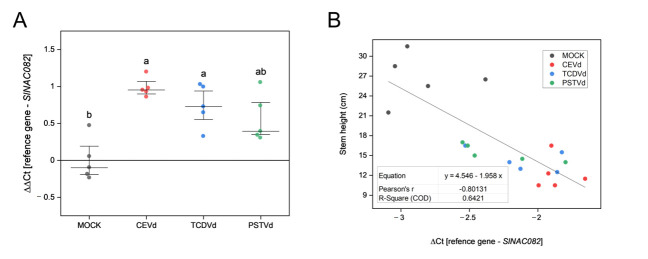
Induction of the ribosomal stress marker *SlNAC082* is related to the severity of the symptoms. (**A**) Real-time PCR analysis of *SlNAC082* expression in agro-inoculated plants with mock, CEVd, TCDVd, and PSTVd at 25 dpi. Expression levels are relative to tomato mock plants and normalized to the tomato actin gene. Data correspond to five independent plants ± SE. Data were analyzed using a Mann–Whitney test and different letters indicate significant differences (*p* ≤ 0.05). (**B**) Correlation value and equation between the relative induction of *SlNAC082* and the decrease in stem length in viroid-inoculated tomato plants.

Ribosomal stress has been described to correlate with viroid symptomatology, it is characterized by a defective processing of rRNA and has been associated to the induction of the *NAC082* gene in *Arabidopsis thaliana* [49]. Since the orthologous gene of *AtNAC082* was previously identified in tomato (accession Solyc11g005920.1.1, Sol Genomics Network, accessed date 7 April 2021) [16,17], we quantified the mRNA levels of tomato *SlNAC082* in agro-inoculated tomato plants, which displayed the most severe symptoms (Figure 2B). The expression of this ribosomal stress marker was upregulated in CEVd- and TCDVd- agro-inoculated tomato plants in comparison with the mock-inoculated control plants (Figure 5A). CEVd was the viroid that caused the highest induction of *SlNAC082*, followed by TCDVd. In order to provide a quantitative value for this apparent correlation between viroid symptoms and induction levels of ribosome stress marker *SlNAC082,* we plotted the decrease in stem height with the relative increase of this gene finding a correlation value of −0.80 for tomato (Figure 5B). Therefore, these data indicate a clear correlation between viroid phenotypic alterations and the induction of ribosomal stress.

Tomato and Arabidopsis NAC082 nucleotide sequences were used to identify the closest gene in eggplant (accession SMEL_000g037840.1.01; Sol Genomics Network, accessed in 10 April 2021 Figure S2), and its expression was analyzed (Figure S3), confirming the induction of *SmNAC082* in the TCDVd- agro-inoculated plants, which displayed the most severe symptoms. Moreover, little or no induction of the ribosome stress marker was observed neither in CEVd- nor in PSTVd- agro-inoculated eggplants, in which viroid levels and symptoms were negligible, as previously shown (Figure 2 and Figure 3). 

**Figure 6:**
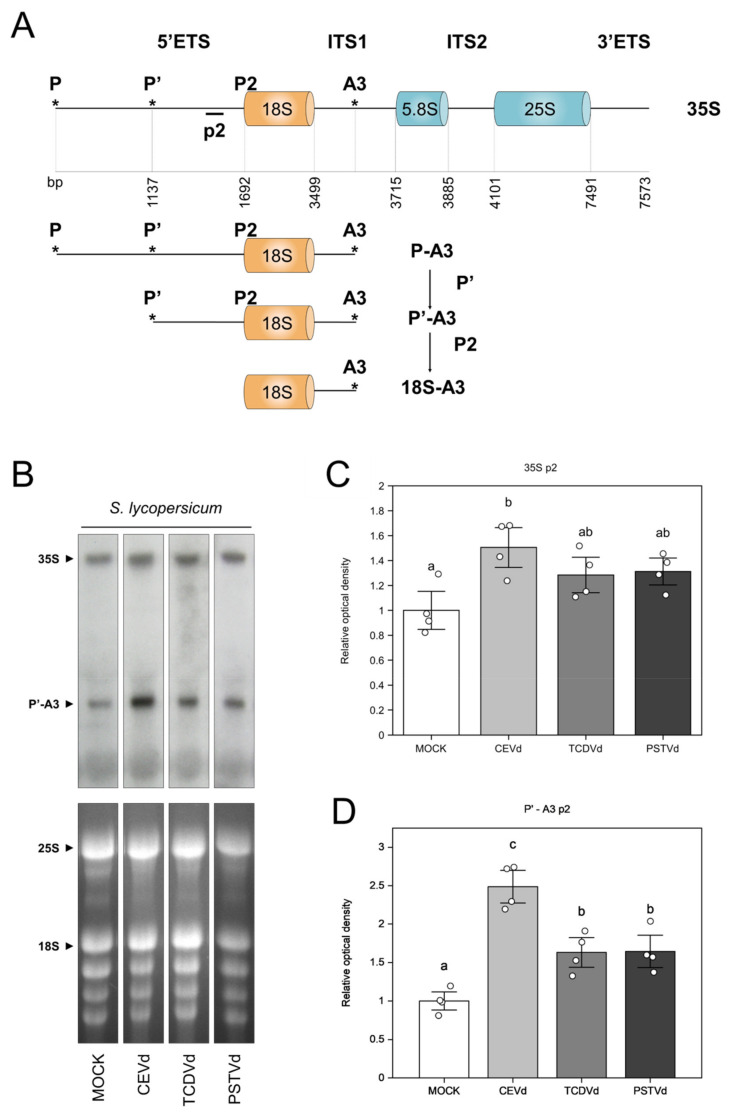
Alterations in *S. lycopersicum* rRNA processing upon agro-inoculation with CEVd, TCDVd, and PSTVd. (**A**) Primary transcript and major 35S pre-ribosomal rRNA processing. Successive cleavages in A3, P’, and P2 and the corresponding P-A3, P’-A3, and 18S-A3 bands are indicated. (**B**) *S. lycopersicum* RNAs were extracted from agro-inoculated mock and infected leaves and hybridized for the detection of pre-rRNA 35S and P’-A3 with the p2 probe. Bottom image corresponds to EtBr-stained gel, and top image corresponds to Northern hybridization. Optical density quantification of the 35S band (**C**) and P’-A3 band (**D**). Data correspond to the mean ± SE of 4 biological replicates and their individual values (white dots). Data were analyzed using a Mann–Whitney test and different letters indicate significant differences (*p* ≤ 0.05).

Ribosomal stress is often caused by impairment in rRNA processing. Thus, we performed a Northern blot analysis with the p2 probe targeting the P’-A3 pre-rRNA (Figure 6A,B), which has been described to be affected upon CEVd infection [16,17]. As Figure 6C,D shows, both 35S and P’-A3 levels were significantly higher in CEVd agro-inoculated plants, which correlates with symptomatology and *SlNAC082* expression levels. 

These results indicate that agro-inoculation with CEVd, TCDVd, and PSTVd impairs ribosome processing in tomato plants, and that this impairment is correlated with disease severity and the activation of the plant defense response (Figure 3 and Figure 4). 

## 3. Discussion

Producing infectious clones of viroids is essential for understanding host-adaptation and disentangling the pathogenesis of these circular RNAs. Specific sequence variations have been linked to viroid diseases [2] and, therefore, infectious clones are required to validate these effects and evaluate viroid fitness. It has been assumed that agrobacterium-mediated viroid-inoculation should be more efficient than inoculation of in vitro generated RNA transcripts because the overall input is expected to be larger as a result of the cDNA expression over time [50]. In fact, agro-inoculation of previously considered non-viable variants of PSTVd revealed that they were indeed infectious but presented very reduced accumulation levels [51] while infection of certain viroid-host combinations, like HSVd-*N. benthamiana* [52], has only been possible by this method. On the other hand, viroid transcripts produced in vitro are required for some experimental approaches such as transfecting protoplast cells [53]. Moreover, *A. tumefaciens* is a plant pathogen by itself and, therefore, in some experiments it might be more desirable to use mechanical inoculation, which supposes an additional stress to the plant [54]. Because each method has its advantages and disadvantages, the aim of this work was to obtain a binary plasmid suitable for both types of inoculation methods. We have effectively generated infectious clones of three viroids of the *Pospiviroid* genus (CEVd, TCDVd, and PSTVd) in an updated binary vector, and assayed their infectivity by agro- and transcript-inoculation in tomato and eggplant.

The bioassays using both inoculation methods in parallel revealed significant phenotypic differences (Figure 2), with the symptomatology in agro-inoculated tomato plants being the most severe and reproducible. CEVd agro-inoculated tomato plants showed a statistically significant reduction of stem height compared to transcript-inoculated plants. Interestingly, eggplant dwarfism was only observed in TCDVd agro-inoculated plants, reinforcing the higher efficiency of the agro-inoculation method (Figure 2A).

Except for CEVd in eggplant, all the other plant-viroid combinations resulted in evident levels of viroid accumulation, regardless of the inoculation method, as revealed by Northern blot hybridization. In fact, CEVd circular form was only detected in one of the eggplant samples at a very low concentration, indicating that this particular CEVd variant is very inefficient in establishing a systemic infection in *Solanum melongena* cv Black beauty. However, it has been previously described that eggplant can be a natural host of CEVd [55] and that important differences in viroid accumulation can occur depending on the plant cultivar [56]. Therefore, it can be hypothesized that the interaction of viroids with specific host factors that may differ between plant varieties should be essential for their spread or replication, while small changes in viroid sequences heavily influence these interactions. 

Moreover, mature circular forms of all three viroids were statistically more abundant in tomato (Figure 3), suggesting that important differences in viroid biogenesis or movement can exist depending on the host, because of the aforementioned aspects of interaction with host factors. Interestingly, we found that the proportion of circular and linear viroid forms varied not only depending on the host (Figure 3), but also with the inoculation method (Figure 3A) which could be attributable to a different progression of the infection. However, there is no strict correlation between the accumulation levels of viroid form and symptoms. For example, even though the accumulation levels of circular and lineal forms of PSTVd were not very different between hosts (Figure 3C), PSTVd disease in tomato is quite severe, while in eggplant, we did not observe any visible alterations (Figure 2A,B, respectively). This lack of correlation between viroid accumulation and symptomatology has also been described by other authors reporting results that vary depending on the specific host-pathogen combinations [17,21,33,57]. 

Given that all viroid-infected tomato plants displayed an evident symptomatology, we decided to further investigate if different viroids might trigger a different signaling defense response, since the disturbance of SA and GA homeostasis has been described in viroid infections [20,21,58,59,60]. In agro-inoculated plants, both total SA and GA were statistically accumulated upon infection with the three viroids, the highest levels corresponding to CEVd-agro-inoculated plants (Figure 4A,B), which correlates to observed symptomatology (Figure 2B) and CEVd accumulation (Figure 3). However, no statistical differences were observed in transcript-inoculated tomato plants with each viroid when compared to mock, with the exception of GA accumulation upon PSTVd- and TCDVd-transcript inoculation. Correlating with the systemic accumulation of these defensive phenolics, the induction of *PR1* was observed in all the agro-inoculated plants, when compared with the mock agro-inoculated plants. The highest induction was produced in CEVd agro-inoculated plants, whilst the highest variability was observed in plants inoculated with transcripts of the same viroid (Figure 4C,D). The high biological variability observed between tested plants due to this inoculation method could explain these results, since mechanical transcript-inoculation is relatively inefficient, and some plants might not be properly infected. Thus, agro-inoculation is proven to be a more efficient and reproducible method for viroid infection.

Here, we report for the first time a symptomatic infection of eggplant by TCDVd. This host-viroid combination has been recently detected in greenhouse conditions without displaying any disease symptoms [32]. That could be explained because of the nucleotide variations between isolates. Given the error-prone replication of viroids, the appearance of new viroid diseases is a constant risk [61], and latent viroid populations in cultivated and non-cultivated species are a reservoir of potentially pathogenic RNAs. This suits perfectly the well-proven relationship between RNA silencing and viroid pathogenesis. 

Moreover, a novel insight in viroid pathogenesis has been the discovery of ribosomal stress in tomato plants infected with CEVd [16,17], the first biotic agent known to cause this defect in ribosome biogenesis. To determine if this alteration is a widespread phenomenon in viroid pathogenesis or if it could be host or viroid specific, we quantitated the induction of orthologous genes of the ribosomal stress marker NAC082 in tomato and eggplant. Our results show that this marker gene was always induced in plants with evident phenotypic alterations, and that the severity of the symptoms was correlated with the level of induction (Figure 5 and Figure S1). Previous research revealed a defect in 18S processing upon CEVd infection [16,17]. We performed a Northern blot hybridization to assess whether rRNA processing was also impaired in tomato agro-inoculated plants (Figure 6). Our results show an impairment in 18S rRNA processing in plants infected provoked by all three viroids, and higher accumulation levels of the unprocessed fragment P’-A3 in CEVd agro-inoculated plants, which correlates with disease severity and plant defense activation. In the light of these findings, ribosomal stress seems to be a novel signature of the diseases caused by pospiviroids. However, the mechanism by which these pathogenic RNAs cause that alteration is yet to be elucidated, as many other aspects of viroid interactions with host factors. 

In conclusion, here we provide a specific tool to produce infectious cDNAs of any viroid which is valid for both inoculation methods (transcript and agro-inoculation). Moreover, the symptoms developed by eggplant and tomato plants agro-inoculated with TCDVd and CEVd, respectively, point out the efficiency of the agro-inoculation method because of a better reproducibility in comparison with transcript-inoculation. Finally, the correlated symptomatology, activation of tomato defense response and ribosomal stress reveals the CEVd-agroinoculation of tomato plants as the most aggressive infection.

## 4. Material and Methods

### 4.1. Binary Vector Construction

The binary vector pMD201t2 was engineered from pMD201t [44] to be suitable for the direct cloning and in vitro/in vivo transcription of dimeric viroid. To that purpose, pMD201t was digested with restriction enzymes *Xho*I and *Hind*III, and the resulting fragment of 6783 bp was excised from a 1% agarose gel and purified using GeneJET Gel Extraction Kit (Thermo Fisher Scientific, Waltham, MA, USA). All restrictions enzymes used are from Thermo Fisher Scientific (Waltham, MA, USA). DNA fragments were amplified with PrimeSTAR HS DNA Polymerase (Takara Bio Inc., Otsu, Shiga, Japan) and ligations were set with an insert: vector ratio of 3:1 and 3U of T4 DNA ligase (Promega, Madison, WI, USA) being transformed into DB3.1 *Escherichia coli* cells. The duplicated promoter CaMV 35S was amplified with Fw 1034 and Rv 1035 primers which include the T7 promoter, and the Popit terminator with Fw 1036 primer which includes the multicloning site and Rv 1037 primer (Table S2). These PCR fragments were digested with *Bsmb*I and a ligation with the lethal gene *ccdB* (excised from pMD201t by *Bsa*I digestion) and its backbone digested with *Xho*I and *Hin*d III was set. The colonies containing the plasmid with a transcriptional unit as depicted in Figure 1A, were selected by chloramphenicol resistance (it is in the *ccdB* transcriptional unit) and colony PCR with Fw T7 promoter and Rv PoPit. The oligonucleotides used are listed in Table S2.

### 4.2. Dimeric Viroid cDNA Construction

CEVd was amplified using SuperScript III One-Step RT-PCR System with Platinum Taq High Fidelity DNA Polymerase (Thermo Fisher Scientific, Waltham, MA, USA) from infected tissue of *Solanum lycopersicum* cv Rutgers. This cultivar of tomato was originally infected with the CEVd isolate M34917.1 [62] in 1998 and, since then, viroid extracts obtained from this cultivar have been recurrently used as inoculum. Monomeric TCDVd cDNA (genebank AF162131.1, accessed in 15 January 2021) was synthesized as a gBlock Gene Fragment (Integrated DNA Technologies, Coralville, IA, USA) while PSTVd RG1 (genebank U23058.1, accessed in 15 January 2021) cDNA was amplified from a pBluescript plasmid provided by J.A. Daròs (IBMCP, Valencia, Spain) [63]. Each viroid sequence was amplified with two sets of primers (Table S2) designed to generate compatible ends upon digestion as described in Figure 1. These DNA fragments were purified and a one-pot reaction was set as follows: 10 U of *Bsa*I, 3 U T4 DNA ligase, 1 mm^3^ of ligase buffer 10× 50 ng of pMD201t and 300 ng of the digested DNA fragments in a final volume of 10 mm^3^. The incubation was performed using a thermocycler with the following conditions: an initial step of 20 min at 310 K, 20 cycles of 1 min at 310 K and 4 min a 289 K, finally holding on the temperature at 289 K until transformation. For each reaction, 1 mm^3^ of the ligation to pMD201t2 was transformed into DH5α electro competent cells and plated onto kanamycin agar plates. Plasmid extraction was performed with GeneJET Plasmid Miniprep Kit (Thermo Fisher Scientific, Waltham, MA, USA) and resulting constructs were sequenced using Rv PoPit and Fw T7 promoter.

### 4.3. In Vitro Transcription 

Viroid transcripts were generated by transcription of 1000 ng of *Xba*I-linearized plasmid with T7 RNA polymerase (Takara Bio Inc., Otsu, Shiga, Japan) for 3 h according to manufacturer instructions. One mm^3^ of each 10 mm^3^ reaction was loaded into a sterile 1% agarose gel, by serial dilutions (0.1, 0.3 and 0.6 mm^3^). RiboRuler High Range RNA Ladder (Thermo Fisher Scientific, Waltham, MA, USA) was used to estimate the RNA concentration, as for the loaded volume (0.83 mm^3^) each ladder band corresponds to 50 ng. 

### 4.4. Plant Material 

*Solanum lycopersicum* cv Rutgers (Western Hybrid Seeds, Hamilton City, CA, USA) and *Solanum melongena* cv Black Beauty (Vilmorin, Alicante, Spain) were used in this work. Seeds were sterilized with a 50% mixture of commercial sodium hypochlorite and Milli-Q H_2_O, and plants were grown in pots with a mixture of vermiculite and peat (1:1) and irrigated with Hoagland solution. Plants were placed in a growth chamber with a 16 h light and 8 h darkness photoperiod and a temperature and relative humidity range of 301 K/297 K and 60%/85% (day/night), respectively. 

### 4.5. Viroid Inoculation

Cotyledons of *Solanum lycopersicum* cv Rutgers and *Solanum melongena* cv Black beauty seedlings were inoculated with dimeric transcripts or agro-infiltrated. For mechanical inoculation, cotyledons were dusted with carborundum (600-mesh), and 4 μg of viroid transcript was placed in each cotyledon and gently rubbed 8–10 times with a sterile glass bar. Agro-inoculation was performed with a culture of *A. tumefaciens* strain C58 harboring the pMD201t2 binary vector with the correspondent viroid-dimer. An overnight grown bacterial culture was diluted in infiltration buffer (MES 0.01 M, MgCl_2_ 0.01 M) up to an optical density of 1 at 600 nm and injected on the abaxial side of one cotyledon using a needle-less syringe. Samples of systemic leaf tissue were collected at 25 days after viroid inoculation.

### 4.6. RNA Extraction and Northern Blot 

Total RNA was extracted from systemic leaves as described previously [64]. For viroid detection, 5 μg of total RNA per sample were loaded into a 5% PAGE prepared in 89 mM TBE and containing 8 M urea [44]. RNA electrophoresis was performed at 200 V for three hours and then RNA was transferred to a nylon membrane at a constant voltage of 10 V. Nucleic acids were cross-linked to the nylon membrane by using ultraviolet light (1 min at 125 millijoules/cm^2^). Hybridisation and chemiluminescent detection were performed as previously described [65].

Northern blots for studying rRNA processing were performed according to Cottilli et al. [15], and p2 probe sequence (5′GAGCGCGGCAGTCATTCGCAAGGAGCATTC3′) was also described by these authors.

### 4.7. Real-Time PCR 

cDNA was synthesized from 1 μg of the extracted RNA using the PrimeScript RT kit and random primers (PerfectReal Time, Takara Bio Inc., Otsu, Shiga, Japan). Quantitative RT-qPCR was carried out as previously described [60] in a 10 mm^3^ volume, using MicroAmpFast 96-Well ReactionPlate (Applied Biosystems, Foster City, CA, USA) plates and PyroTaq EvaGreen qPCR Master Mix (CMB, Madrid, Spain) in a QuantStudio 3 Real-Time PCR System, 96-well, 0.1 cm^3^ (Applied Biosystems, Foster City, CA, USA). Actin was used as the endogenous gene of reference for *Solanum lycopersicum* and cyclophilin for *Solanum melongena* [66]. The oligonucleotides used are listed in Table S2.

### 4.8. Extraction and Analysis of SA and GA

Levels of free and total SA and GA were analyzed in methanol extracts from leaf tissues of viroid-inoculated tomato and eggplants and their corresponding mock-inoculated controls at 25 dpi. Homogenized samples (0.5 g fresh weight) were extracted using 2 cm^3^ 100% methanol (containing 25 mM o-anisic acid as internal standard) and ultrasounds during 10 min. After centrifugation, the supernatant was divided in two equal parts and dried under a flow of nitrogen at 313 K. To analyze total SA and GA, one half of the dried residue was submitted to an enzymatic hydrolysis employing 10 U of almond β-glycosidase (EC3.2.1.21) (14.3 U/mg) (Thermo Fisher Scientific, Waltham, MA, USA) in 100 mm^3^ of water and then resuspended in 900 mm^3^ of 50 mM sodium acetate (pH 4.5). To analyze free SA and GA, the other half was resuspended in 900 mm^3^ of 50 mM sodium acetate (pH 4.5) and 100 mm^3^ of water. The reactions were incubated overnight at 310 K and then stopped by adding 75 mm^3^ of 70% perchloric acid (5% (*v*/*v*) final concentration) and maintained at 277 K for 1 h. After centrifugation at 14000× *g* for 15 min, the supernatants were extracted with 2.5 cm^3^ of cyclopentane/ethylacetate (1:1, *v*/*v*). The organic upper phase was dried under a flow of nitrogen at 313 K, resuspended in 300 mm^3^ of methanol and filtered through 0.45 μm Minispike nylon filters (Waters, Milford, MA, USA) to HPLC analysis. An aliquot of 30 mm^3^ was injected with a Waters 717 autosampler into an analytical reverse-phase C18 column (5 μm pore size, 4.6 mm ×150 mm) (Scharlab, Barcelona, Spain) maintained at room temperature, and equilibrated with 1% acetic acid in water. A 20 min linear gradient of 1% (*v*/*v*) acetic acid to 100% methanol was applied at a flow rate of 1 cm^3^/min with a 1525 Waters Binary HPLC pump. SA and GA were detected by a 2475 Waters Multi λ Fluorescence Detector (λ excitation 313 nm and λ emission 405 nm) and quantified using the Waters Empower software by comparing retention times with those of authentic standards. Final data were obtained by correcting for losses in the extraction procedure using the internal standard.

### 4.9. Statistical Analysis

IBM SPSS Statistics 27 software was used for all statistical analysis. A *p* ≤ 0.05 was considered as statistically significant. The Shapiro–Wilk test was used for sample normality. The Mann–Whitney test was used to compare two independent non-parametric samples. A multiple group non-parametric comparison was performed by using a Kruskal–Wallis test.

## Data Availability

The data presented in this study are available in this article and Supplementary Materials.

## Supplementary Materials

The following are available online at https://www.mdpi.com/article/10.3390/ijms22126189/s1.
