# Supplementary material for: Symptom Severity, Infection Progression and Plant Responses in Solanum Plants Caused by Three Pospiviroids Vary with the Inoculation Procedure
Source: Int J Mol Sci. 2021 Jun 8;22(12):6189. doi: 10.3390/ijms22126189 (PMC8273692; doi:10.3390/ijms22126189)
Supplement: Supplementary file 1 [file ijms-22-06189-s001.zip › suppl tables.pdf]

Scheme 1. Occurrence in viroid sequences of restriction enzymes recognition sites present in pMD201t2 for linearization before in vitro transcription.

	RECOGNITION SITE	OCURRENCE	PERCENTAGE
*Bcu*I	ACTAGT	238	2.1%
*Bam*HI	GGATCC	1781	15.7%
*Eco*RI	GAATTC	2174	19.1%
*Bgl*II	AGATCT	47	0.4%
	*Bcu*I/*Bam*HI/*Eco*RI/*Bgl*II	0	0.0%

Supplementary Table 2. List of oligonucleotides used for binary vector construction, assembly of viroid-cDNA dimers and real-time PCR.

**Binary Vector Construction and Sequencing**	**Sequence 5′-3′**
Fw 1034	CGTCTCAAGCTTACTCGAGCTCGGG
Rv 1035	CGTCTCCCATGGCCCCTATAGTGAGTCGTATTATACCGATCCTGGA AGTATTTG
Fw 1036	CGTCTCTCTAGACTAGTGGATCCGAATTCAGATCTGACCCTAGACT TGTCCATC
Rv 1037	CGTCTCCTCGAGGTATATGGAATTGGTTAATAAAATGC
Fw T7 promoter	TAATACGACTCACTATAGGG
Rv PoPit	GTTGGCCAATCCAGAAGATGG
**Assembly of viroid-cDNA dimers**	**Sequence 5′-3′**
Fw1 CEVd	GGTCTCCCATGCGGGATCTTTCTTGAGGTTCC
Rv2 CEVd	GGTCTCGAGGGACCCAATCTAGGGTTC
Fw3 CEVd	GGTCTCCCCCTCGGGATCTTTCTTGAGGTTCC
Rv4 CEVd	GGTCTCGCTAGAGGGACCCAATCTAGGGTTC
Fw1 PSTVd	GGTCTCCCATGGCGGAACTAAACTCGTGGTTC
Rv2 PSTVd	GGTCTCCAGGAACCAACTGCGGTTCC
Fw3 PSTVd	GGTCTCTTCCTCGGAACTAAACTCGTGGTTC
Rv4 PSTVd	GGTCTCGCTAGCAGGAACCAACTGCGGTTCC
Fw1 TCDVd	GGTCTCACATGCGGAACTAAACTCGTGGTTCCT
Rv2 TCDVd	GGTCTCTAGGAACCAACTGCGGTTCC
Fw3 TCDVd	GGTCTCTTCCTCGGAACTAAACTCGTGGTTC
Rv4 TCDVd	GGTCTCACTAGAGGAACCAACTGCGGTTC
**Real-time PCR**	**Sequence 5′-3**
Fw *Cyclophilin*	GCGCCAAATTCAAGGACGAGAACT
Rv *Cyclophilin*	ACAGCCTCGGCCTTCTTAATCACA
Fw *SmNAC082*	TTCACCTAGGTCATCCCCCGCG
Rv *SmNAC082*	GGACCCGGTCCACTCTTCTGGA

Fw *Actin 4*	CAAGGGTGGGTTCGCAGGAGATGATGC
Rv *Actin 4*	GTCTTTTTGACCCATACCCACCATCACAC
Fw *SlNAC082*	TGCTGAAACCATTGGAACTG
Rv *SlNAC082*	CCAAGGAATTGCTTCCAAAA
Fw *PR1*	ACTCAAGTAGTCTGGCGCAACTCA
Rv *PR1*	AGTAAGGACGTTGTCCGATCGAGT
