# Supplementary figures and images for: Symptom Severity, Infection Progression and Plant Responses in Solanum Plants Caused by Three Pospiviroids Vary with the Inoculation Procedure
Source: Int J Mol Sci. 2021 Jun 8;22(12):6189. doi: 10.3390/ijms22126189 (PMC8273692; doi:10.3390/ijms22126189)

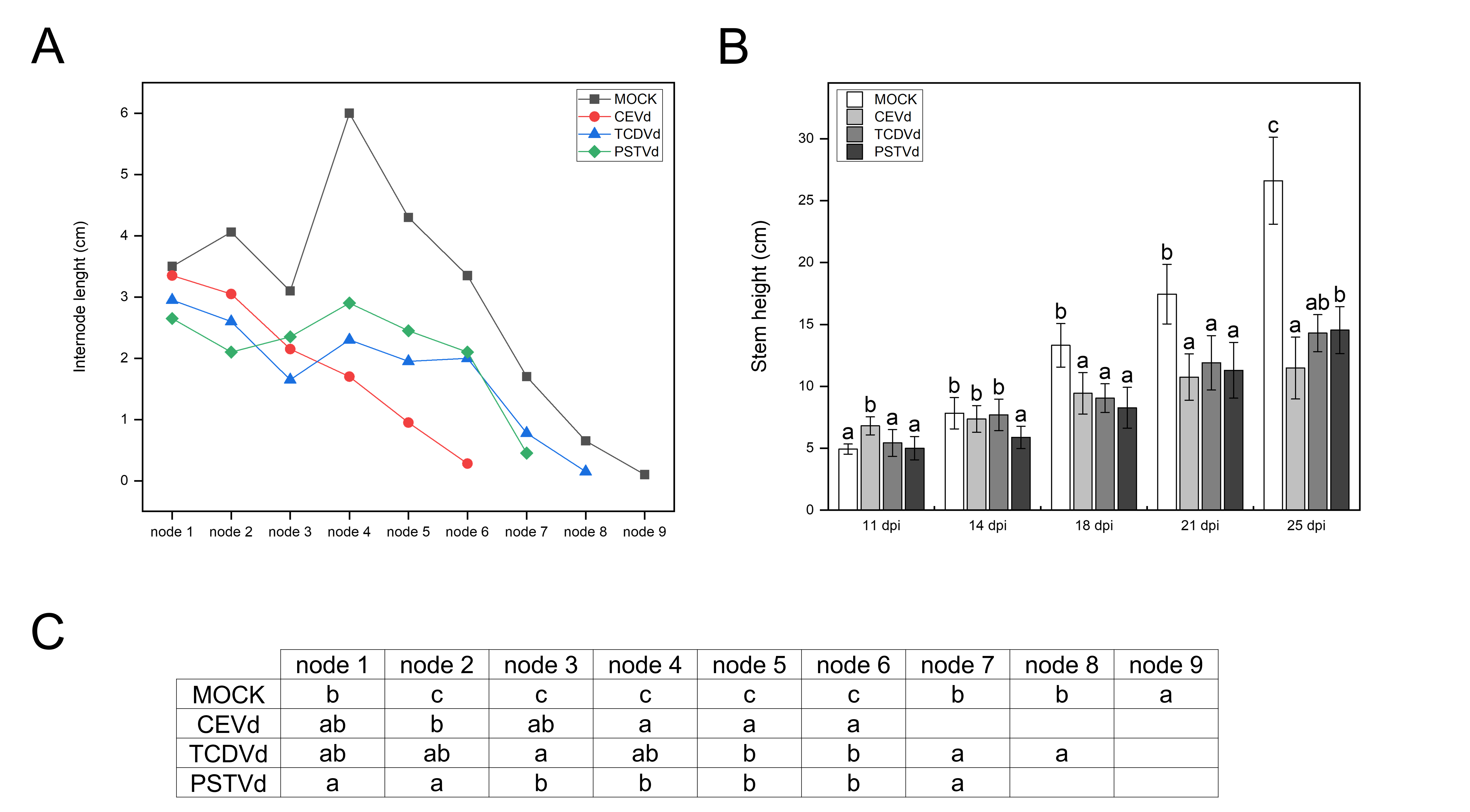

Supplement: Supplementary file 1 [file ijms-22-06189-s001.zip › Figure S1.png]

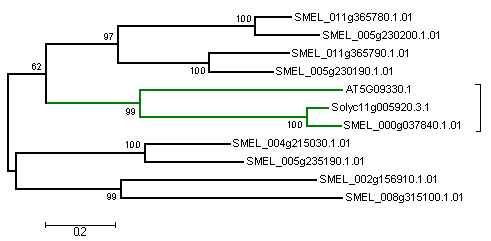

Supplement: Supplementary file 1 [file ijms-22-06189-s001.zip › Figure S2.png]

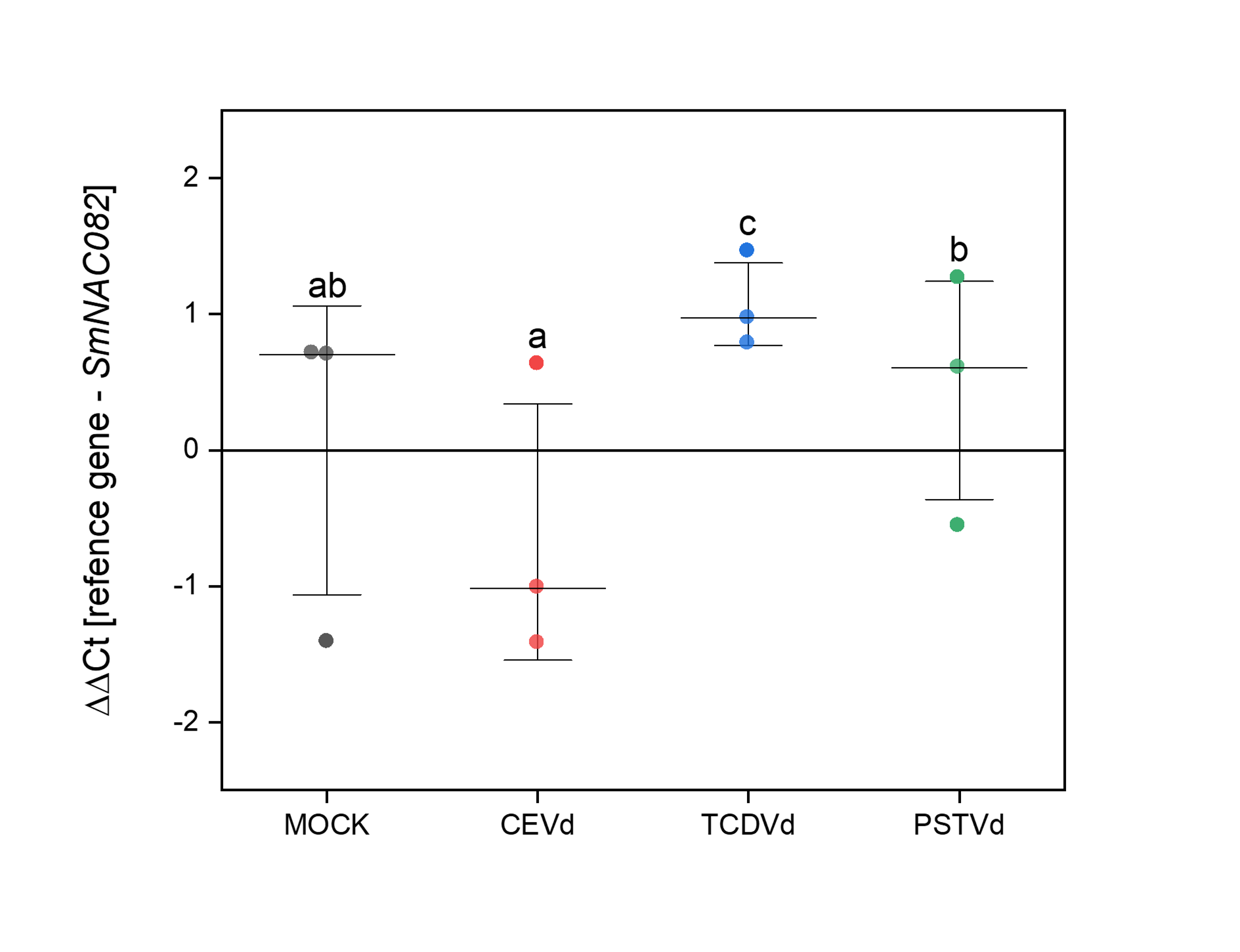

Supplement: Supplementary file 1 [file ijms-22-06189-s001.zip › Figure S3.png]
